# Testicular macrophages: core immune regulators and emerging therapeutic targets in spermatogenic dysfunction

**DOI:** 10.3389/fimmu.2026.1868707

**Published:** 2026-06-23

**Authors:** Zesong Jiang, Xiaohe Xuan, Zhongjian Qiu, Xiang Zhou, Hui Ma, Zhiguo Zhu

**Affiliations:** 1 School of Clinical Medicine, Jining Medical University, Jining, Shandong, China; 2 Department of Urology, Affiliated Hospital of Jining Medical University, Jining Medical University, Jining, Shandong, China; 3 Reproductive Medicine Center, The Third Affiliated Hospital of Guangxi Medical University, Nanning, Guangxi, China; 4 Women and Children’s Hospital, Qingdao University, Qingdao, Shandong, China

**Keywords:** macrophage plasticity, non-obstructive azoospermia, single-cell RNA sequencing, spermatogenic dysfunction, testicular macrophages

## Abstract

Spermatogenic dysfunction is one of the leading causes of male infertility, mainly including non-obstructive azoospermia (NOA) and abnormalities in sperm count or quality. As an immune-privileged organ, the testis relies heavily on immune homeostasis to sustain spermatogenesis, with testicular macrophages playing a central regulatory role in this process. Under normal physiological conditions, testicular macrophages predominantly exhibit immune tolerance-related phenotypes, regulate local inflammatory responses, maintain immune privilege, and contribute to hormonal homeostasis, all of which collectively support spermatogenesis. However, during spermatogenic dysfunction, significant alterations occur in the number, distribution, functional status, and signaling pathways of testicular macrophages. Aberrant activation of inflammation-related signaling pathways in macrophages and imbalance in immune regulation can induce chronic low-grade inflammation, disrupt the spermatogenic microenvironment, and further exacerbate spermatogenic dysfunction. In recent years, with the advancement of reproductive immunology and single-cell RNA sequencing technologies, numerous studies have highlighted the critical role of testicular macrophages in spermatogenic dysfunction and explored potential therapeutic strategies targeting these cells. Nevertheless, the existing body of research is fragmented, lacking systematic reviews and comprehensive integration. This review aims to provide a comprehensive synthesis of the developmental origins and functional heterogeneity of testicular macrophages and to discuss their mechanistic roles in various male spermatogenic dysfunctions. Furthermore, we review the current therapeutic advances targeting testicular macrophages and discuss the main challenges and future research directions in this field. Our study provides theoretical insights for the mechanistic study of male spermatogenic dysfunction and the development of precision intervention strategies.

## 1 Introduction

Infertility impacts 10% to 15% of couples around the world, with male factors responsible for half of the instances (1). Spermatogenic dysfunction is a major cause of male infertility. Spermatogenic dysfunctions can be broadly classified into non-obstructive azoospermia (NOA) and abnormalities in sperm morphology or number. NOA represents the most severe form of spermatogenic dysfunction, characterized by impaired intratesticular spermatogenesis and the complete absence of spermatozoa in the ejaculate (2). Abnormalities in sperm morphology or number include oligozoospermia, asthenozoospermia, teratozoospermia, as well as combined forms such as oligoasthenoteratozoospermia (3). Although these conditions do not result in complete azoospermia, severe cases can still markedly impair fertility and may even progress to NOA. Obstructive azoospermia (OA) is primarily caused by obstruction of the reproductive tract, which prevents sperm release (4). Pathophysiologically, it is generally not considered a primary spermatogenic dysfunction. However, long-term obstruction of the spermatic ducts may also have secondary effects on testicular spermatogenesis, leading to abnormal sperm production. In recent years, male sperm quality has shown a continuous global decline influenced by genetic, hormonal, environmental, and lifestyle factors (5). Studies show that over the past few decades, average sperm quality has declined by approximately 50%, while the annual incidence of male infertility has increased by about 0.291% per year (6, 7).

Testicular macrophages play an indispensable role in maintaining male reproductive function. The testis is an immune-privileged organ requiring tolerance to germ cells bearing self-antigens, with testicular macrophages serving as critical effectors of this process (8). In the physiological state, testicular macrophages mainly display an immunosuppressive M2-like phenotype and contribute to immune tolerance by producing high levels of anti-inflammatory cytokines (e.g., IL-10 and TGF-β) while maintaining low expression of pro-inflammatory cytokines (e.g., TNF-α and IL-12) (9, 10). M2-like macrophages also promote regulatory T cell differentiation and inhibit pro-inflammatory nuclear factor kappa B (NF-κB) signaling, thereby protecting spermatogenesis from immune attack during inflammatory stress (11, 12). However, in disease states, testicular macrophages may transition from maintaining fertility to contributing to the development of diverse spermatogenic abnormalities. Aberrant accumulation and phenotypic–functional dysregulation of testicular macrophages critically contribute to chronic low-grade inflammation, breakdown of immune privilege, disruption of hormonal homeostasis, and deterioration of the spermatogenic niche (13, 14). Studies have shown that patients with spermatogenic dysfunction exhibit a significant increase in macrophage infiltration and activation of inflammation-related signaling pathways in their testicular tissues (11, 15, 16). These findings suggest that testicular macrophages may exist in an abnormal and complex state of immune imbalance and may serve as a key pathological factor that reshapes the inflammatory testicular microenvironment and drives the development and progression of spermatogenic dysfunction.

Consequently, modulating the dysregulated states of macrophages has emerged as a potentially promising therapeutic strategy for spermatogenic dysfunction. Recent advancements in reproductive immunology and single-cell RNA sequencing (scRNA-seq) technologies have significantly enhanced our understanding of the role of testicular macrophages in the regulation of spermatogenesis and their contribution to spermatogenic dysfunction. Despite the growing body of primary studies that have emerged in recent years, there remains a lack of comprehensive reviews that systematically address the origins, functional heterogeneity, and roles of testicular macrophages in various forms of spermatogenic dysfunctions. In this review, we provide a comprehensive overview of the developmental origins and functional features of testicular macrophages, with an emphasis on their roles in NOA, OA, and sperm parameter abnormalities. In addition, we summarize current progress and future directions in macrophage-targeted therapies for spermatogenic dysfunction, aiming to provide theoretical insights into its immunological underpinnings and potential therapeutic interventions.

## 2 ScRNA-seq redefines testicular macrophage origin and classification

**Figure 1:**
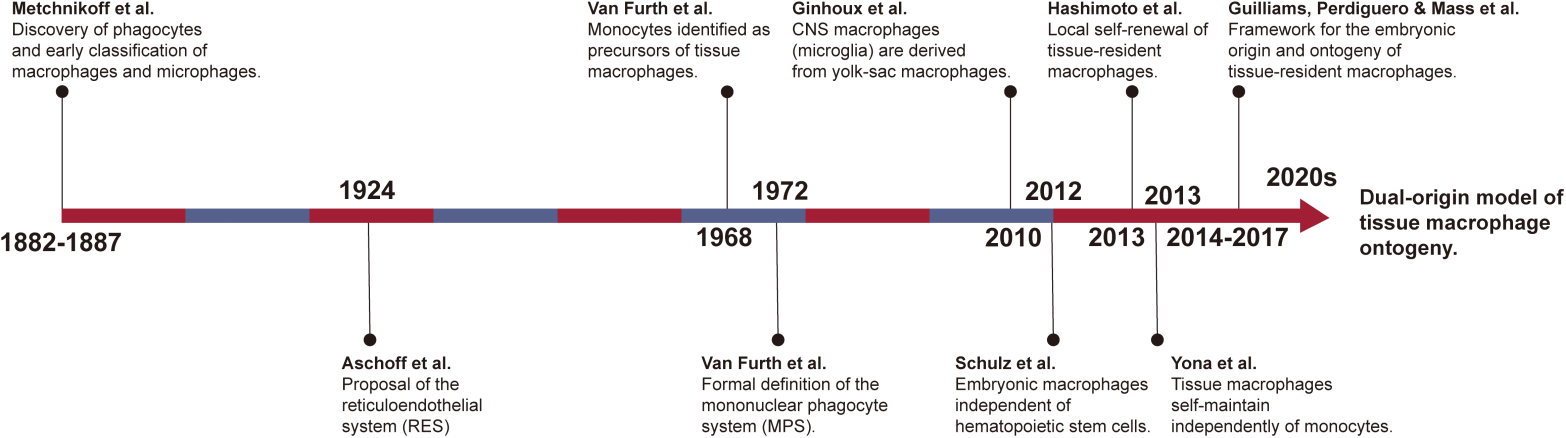
A timeline of key milestones shaping to dual-origin model of macrophages. This timeline summarizes major milestones in the understanding of macrophage origin, highlighting the transition from the classical monocyte-derived model to the currently accepted dual-origin model. CNS, Central Nervous System.

In 1968, van Furth et al. (17) proposed that circulating blood monocytes are the precursors of tissue macrophages, and in 1972 formally introduced the concept of the mononuclear phagocyte system. This theory posits that tissue macrophages originate from hematopoietic stem cells (HSCs) located in the bone marrow (18). Initially, HSCs differentiate into monocytes, which then enter the circulatory system and migrate to diverse tissues throughout the body. Within these tissues, monocytes further differentiate into macrophages, characterized by a limited capacity for proliferation. These macrophages are consistently replenished and renewed by monocytes circulating in the bloodstream. However, the widespread use of scRNA-seq has fundamentally reshaped our understanding of macrophage ontogeny (19). Traditional transcriptomic approaches (e.g., bulk RNA sequencing), typically analyze pooled populations of thousands of cells, thereby providing only averaged gene expression profiles (20, 21). In macrophage research, this approach can obscure the transcriptional signatures of distinct macrophage subsets, as well as those of other immune cell types, making it difficult to accurately define their cellular identities and developmental origins. In contrast, scRNA-seq enables the isolation and independent analysis of individual cells, thereby facilitating the unbiased identification of diverse macrophage subsets (22). Moreover, the integration of pseudotime analysis, genetic fate mapping, and spatial transcriptomics enables the reconstruction of cellular trajectories at single-cell resolution, thereby redefining our understanding of macrophage ontogeny (19, 23, 24). In 2010, Ginhoux et al. (25) demonstrated through fate mapping analysis that certain macrophages in the central nervous system originate from primitive yolk sac–derived macrophages. In 2012, Schulz et al. (26) showed that a subset of tissue macrophages arises from embryonic yolk sac progenitors and develops independently of HSCs. In 2013, Yona et al. (27) demonstrated that many tissue macrophages are established before birth and maintained independently of circulating monocytes under homeostatic conditions. In the same year, Hashimoto et al. (28) further showed that these tissue macrophages are capable of local self-renewal. Based on these findings, a dual-origin model of macrophage ontogeny has gradually emerged and is now widely accepted (**Figure 1**). According to this model, macrophages arise from two distinct sources: tissue-resident macrophages and monocyte-derived macrophages. Tissue-resident macrophages originate from yolk sac or fetal liver progenitors, seed tissues during embryonic development, and are maintained in adulthood through local self-renewal (29). In contrast, monocyte-derived macrophages originate from HSCs and are continuously replenished by circulating monocytes.

**Figure 2:**
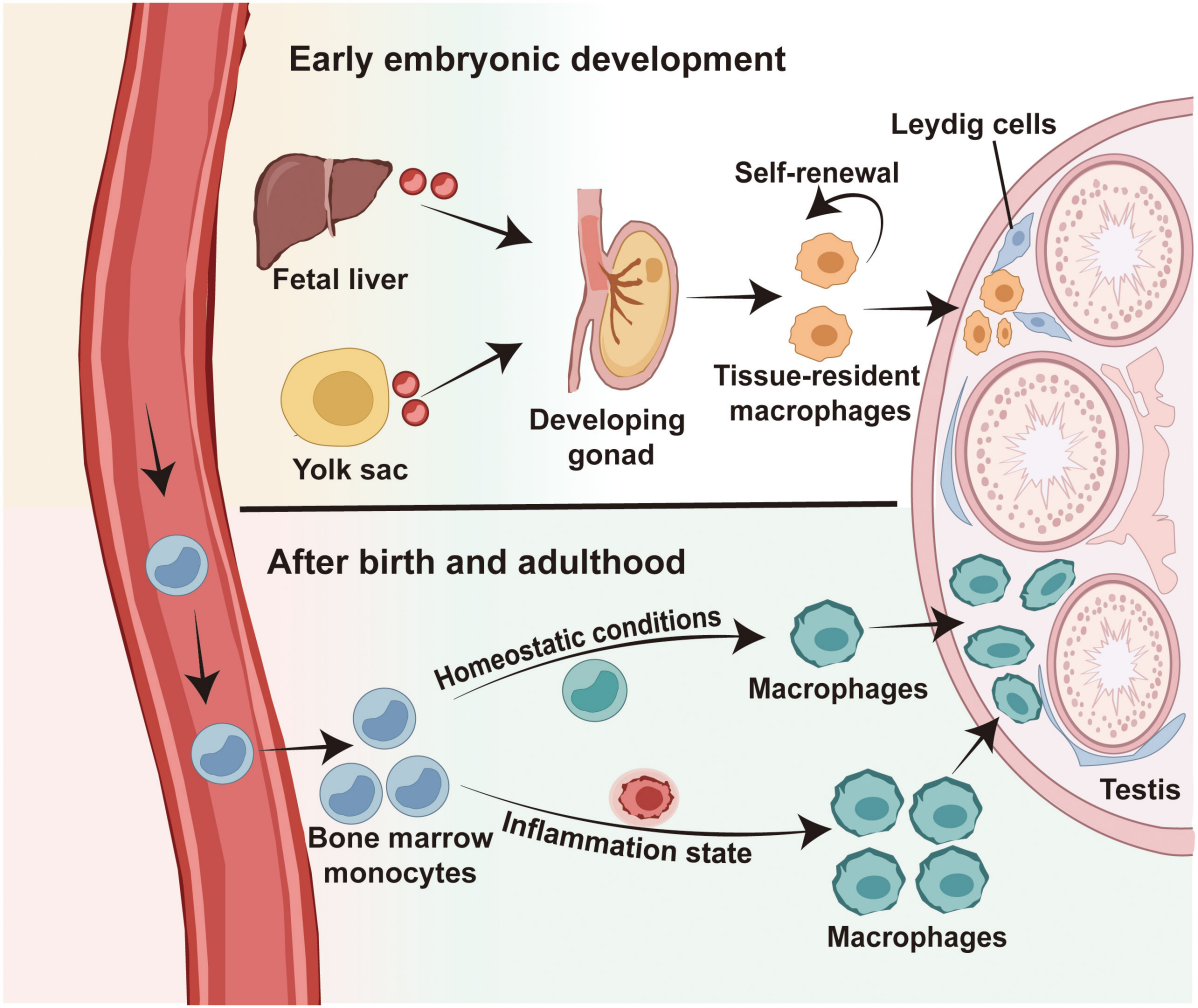
Dual origin and maintenance of testicular macrophages. Tissue-resident macrophages arise from yolk sac or fetal liver progenitors and are maintained by self-renewal, whereas monocyte-derived macrophages are replenished by circulating monocytes. Under inflammatory conditions, monocyte recruitment and macrophage differentiation are markedly increased.

Testicular macrophages represent a classic example of the dual-origin model of macrophages (**Figure 2**). During early embryonic development, hematopoietic progenitor cells migrate to and seed the undifferentiated fetal gonad (30). Traditionally, these progenitors were thought to originate primarily from the yolk sac; however, recent studies by Lokka et al. (31) suggest that the fetal liver represents their main source. Within the testis, these progenitors differentiate into macrophages, establishing the initial pool of tissue-resident macrophages with self-renewal capacity (32, 33). These macrophages play a crucial role in testicular morphogenesis. By secreting angiogenic factors, such as vascular endothelial growth factor, they guide blood vessel growth toward specific regions, which is essential for the formation of testis cords, the distribution of interstitial cells, and the establishment of functional compartmentalization (32). Notably, these macrophages are maintained after birth primarily through local proliferation, with minimal dependence on bone marrow-derived monocytes. Even following macrophage depletion, bone marrow–derived cells are inefficient at replenishing this niche (28). Under homeostatic conditions, tissue-resident macrophages may secrete regulatory factors such as IL-10 and TGF-β to suppress the excessive activation of infiltrating monocyte-derived macrophages, thereby preventing detrimental autoimmune or inflammatory damage and promoting the rapid restoration of tissue homeostasis following pathogen clearance (34, 35). After birth and into adulthood, testicular macrophages can be supplemented by monocytes derived from bone marrow HSCs (36). Under physiological homeostasis, this contribution is minimal and occurs at a very low rate (37). However, during testicular inflammation, infection, or physical injury, circulating monocytes are robustly recruited to the affected sites by chemotactic signals (30, 38). These newly recruited monocytes rapidly differentiate into macrophages and serve as key effectors of the acute immune response. Compared with tissue-resident macrophages, monocyte-derived macrophages exhibit a heightened pro-inflammatory activation potential and elevated expression of inflammatory cytokines. They are primarily responsible for pathogen clearance and antigen presentation to activate adaptive immunity, but generally have a shorter lifespan (39). These two macrophage populations coexist long-term in the adult testis and establish a division of labor along with functional crosstalk.

**Figure 3:**
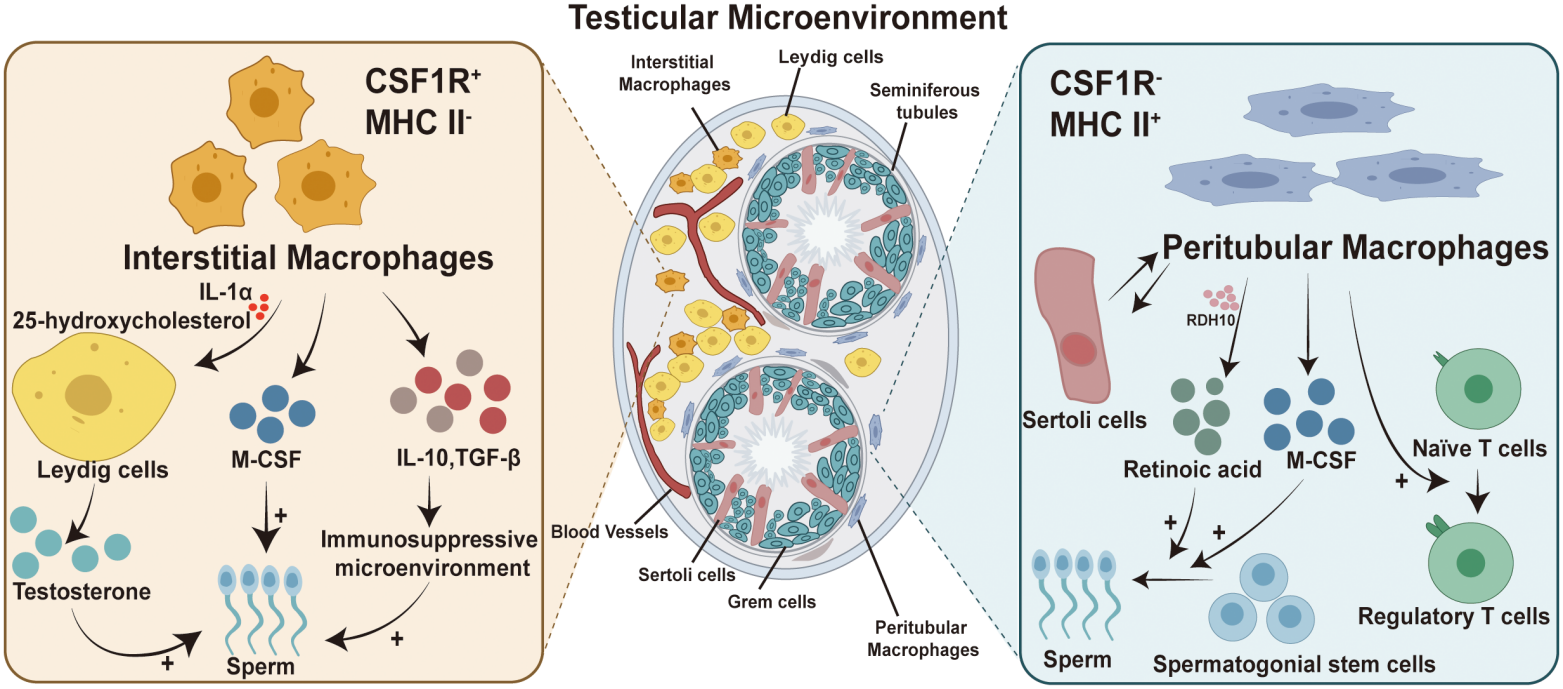
Functional differences between interstitial and peritubular macrophages. Interstitial macrophages promote Leydig cell proliferation and differentiation and stimulate testosterone production by secreting IL-1α and 25-hydroxycholesterol. They also maintain an immunosuppressive testicular microenvironment through high expression of anti-inflammatory cytokines such as IL-10 and TGF-β, and support spermatogenesis via macrophage colony-stimulating factor (M-CSF) secretion. In contrast, peritubular macrophages express retinol dehydrogenase 10 (RDH10) and M-CSF, induce the differentiation of naïve T cells into regulatory T cells. In addition, peritubular macrophages regulate Sertoli cell function through the secretion of cytokines and maintain local immune tolerance, thereby promoting the stability of the blood–testis barrier. M-CSF, macrophage colony-stimulating factor; RDH10, dehydrogenase 10.

In traditional perspectives, testicular macrophages were regarded as a predominantly homogeneous population, characterized by their interstitial localization and a limited set of surface markers, such as F4/80 (11). More recent scRNA-seq studies have utilized classical pan-macrophage enrichment markers, like CD68 and CD74, to better resolve the heterogeneity of the testicular macrophage population (40, 41). Then, two principal subsets of testicular macrophages were identified by scRNA-seq and subsequently defined according to their anatomical localization: interstitial macrophages (CSF1R^+^, MHC II^-^) and peritubular macrophages (CSF1R^-^, MHC II^+^) (42) (**Figure 3**). Interstitial macrophages undergo differentiation at an early developmental stage and are present in the testicular interstitium at birth (33). These macrophages are intimately associated with Leydig cells and specifically facilitate testosterone production, thereby playing a crucial role in supporting the hormonal microenvironment. The differentiation of peritubular macrophages occurs later compared to interstitial macrophages, beginning between the late fetal and early postnatal stages (35). Their numbers progressively increase in conjunction with testicular development, reaching functional maturity by late puberty. Situated around the seminiferous tubules, peritubular macrophages are integral to the formation of the spermatogonial stem cell niche and play a crucial regulatory role in spermatogenesis, particularly influencing the proliferation and differentiation of spermatogonia (33). Additionally, several rare macrophage subtypes have been discovered, including osteoclast-like macrophages (SIGLEC15^+^) and microglia-like macrophages (TREM2^+^), which are implicated in the regulation of vascular development and early testicular morphogenesis (43). Recent high-resolution studies demonstrate that the two major macrophage subsets in the testis—interstitial macrophages and peritubular macrophages—conform to the dual-origin model of macrophages. Both populations are predominantly derived from embryonic progenitors and complete tissue colonization before birth or during the perinatal period (31, 36). Postnatally, under physiological homeostasis, these cells are primarily maintained through local self-renewal. Under pathological conditions, they can be replenished by circulating monocytes from the blood. These subsets do not exist in isolation; rather, through their distinct functional specializations, they collectively establish a highly coordinated immune microenvironment that supports spermatogenesis.

## 3 Heterogeneity and function of testicular macrophages

### 3.1 Interstitial macrophages

The primary role of interstitial macrophages is to facilitate testosterone synthesis by Leydig cells (44). These macrophages and Leydig cells establish specialized structures marked by interdigitating cytoplasmic processes, which allow for intimate cell–cell interactions (31). Typically, each interstitial macrophage is associated with approximately four to eight Leydig cells (35, 45). By secreting IL-1α and 25-hydroxycholesterol, interstitial macrophages directly enhance the proliferation and differentiation of Leydig cells and stimulate the production of testosterone (11, 46). Interestingly, Duan et al. (47) reported that mouse interstitial macrophages express multiple steroidogenic enzymes and may possess a limited capacity for autonomous testosterone production. However, as this observation is currently based on a single study and differs from the conventional view that steroidogenesis is largely restricted to Leydig cells, these findings still require further independent validation. Concurrently, interstitial macrophages play a crucial role in establishing an immune-tolerant microenvironment. This function is manifested in two main aspects. First, they highly express and secrete anti-inflammatory cytokines, including IL-10 and TGF-β, and represent the principal source of local immunosuppressive cytokines in the testis. Second, under homeostatic conditions, their pro-inflammatory signaling pathways (e.g., the Toll-like receptor and NF-κB pathways) exhibit low activity, which contributes to the maintenance of testicular immune privilege (10, 34, 35, 48). In addition, interstitial macrophages express macrophage colony-stimulating factor (M-CSF) (49). M-CSF functions as a direct supportive element for spermatogenesis by activating the colony-stimulating factor-1 (CSF-1)/colony-stimulating factor-1 receptor (CSF-1R) signaling pathway, which in turn facilitates the self-renewal, proliferation, and differentiation of spermatogonial stem cells (50, 51). A deficiency in M-CSF leads to a significant decrease in the number of spermatogonial stem cells and results in compromised spermatogenesis.

### 3.2 Peritubular macrophages

Peritubular macrophages play a crucial role in the formation and maintenance of the spermatogonial stem cell niche. These macrophages highly express retinoic acid–synthesizing enzymes, notably retinol dehydrogenase 10 (RDH10), facilitating the production of retinoic acid within the peritubular microenvironment of the seminiferous tubules (30). Retinoic acid directly aids spermatogonial stem cell differentiation and meiosis initiation, thus facilitating spermatogenesis (52). Peritubular macrophages also secrete M-CSF, which further contributes to the regulatory mechanisms governing spermatogonial stem cells. Furthermore, peritubular macrophages exhibit roles in immune surveillance and the induction of immune tolerance. These cells envelop the seminiferous tubules and are strategically positioned outside the blood–testis barrier (BTB), adjacent to the basement membrane (53). This localization situates them as the initial line of immune defense, facilitating the direct detection of signals from the interstitial space and the early monitoring of germ cell antigens that may “leak” due to BTB disruption. Peritubular macrophages demonstrate elevated expression of genes involved in antigen processing and presentation, such as H2-DMb, H2-Eb1, Nlrp3, H2-K1, and Icam1, which provides a molecular basis for the presentation of captured antigens to T cells (33). Notably, although peritubular macrophages are capable of antigen presentation, their primary function is to promote antigen-specific immune tolerance rather than immune activation. Through the presentation of self-antigens, they facilitate the differentiation of naïve T cells into regulatory T cells or promote T-cell anergy, thereby mitigating autoimmune responses against germ cells (35, 40, 54). This mechanism is crucial in preventing immune-mediated attacks on germline cells and in maintaining the immune privilege of the testicular environment. Overall, the two macrophage populations fulfill complementary functions in preserving testicular immune privilege. Interstitial macrophages primarily contribute to the establishment of a broadly immunosuppressive microenvironment through the secretion of anti-inflammatory mediators, whereas peritubular macrophages are involved in the facilitation of precise, antigen-specific immune regulation.

Peritubular macrophages are closely linked to Sertoli cells, which crucially regulate the recruitment of precursor cells for peritubular macrophages (55). Evidence shows that the targeted depletion of Sertoli cells at specific stages of fetal development in mice leads to a marked reduction in the population of testicular monocytes (36). Sertoli cells additionally regulate differentiation and spatial positioning of peritubular macrophage. After disruption of Sertoli cell identity, peritubular macrophage differentiation is impaired, and these cells fail to properly localize around the seminiferous tubules, instead ectopically accumulating in the interstitial compartment (23). Moreover, Sertoli cells modulate the phenotype of peritubular macrophages. *In vitro* co-culture experiments indicate that Sertoli cells facilitate the M2 polarization of peritubular macrophages, as demonstrated by an elevated proportion of CD206^+^ macrophages (56). *In vivo* studies further reveal that the transplantation of Sertoli cells augments the expression of the immunosuppressive receptor Mer tyrosine kinase on testicular macrophages (both interstitial and peritubular macrophages), encompassing both interstitial and peritubular subsets, thereby aiding in the regulation of inflammation (57). Conversely, dysfunctions in macrophages also can adversely affect Sertoli cell function and compromise the integrity of the BTB. Although Sertoli cells are in close association with peritubular macrophages, caution is warranted when attributing these effects to specific macrophage subsets. Li et al. (58) showed that BTB disruption and Sertoli cell dysfunction are associated with M1-like macrophage activation. Yi et al. (59) reported that peritubular macrophages increase around seminiferous tubules following toxicant-induced disruption of the BTB, indicating their involvement in tubular damage responses. However, current evidence has not established that peritubular macrophages are the principal source of pro-inflammatory mediators driving Sertoli cell dysfunction and BTB breakdown. Macrophage-mediated Sertoli cell and BTB impairment may involve multiple macrophage subsets rather than resident peritubular macrophages alone.

### 3.3 M1-like and M2-like macrophage polarization

**Figure 4:**
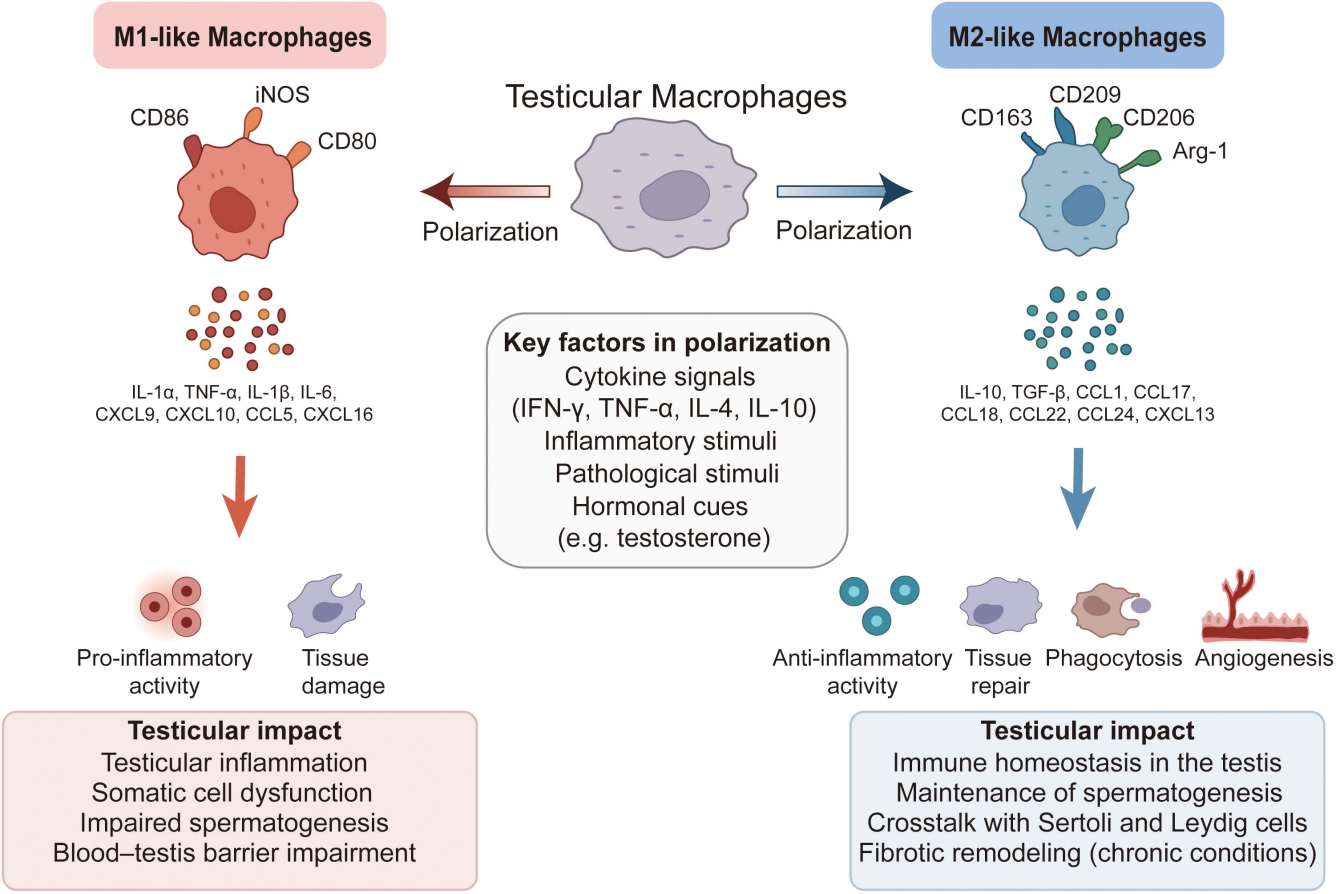
Dynamic polarization of macrophages: M1-like and M2-like phenotypes. Macrophages exhibit dynamic polarization between M1-like and M2-like macrophages. M1-like macrophages produce pro-inflammatory cytokines and contribute to inflammatory responses and tissue damage, whereas M2-like macrophages secrete anti-inflammatory mediators and promote tissue repair, phagocytosis, and angiogenesis.

Macrophage functional states exhibit a high degree of plasticity and have traditionally been broadly classified into two polarization phenotypes: pro-inflammatory M1-like macrophages and immunosuppressive M2-like macrophages (**Figure 4**) (60). M1-like macrophages are characterized by high expression of iNOS and the robust production of pro-inflammatory cytokines, including TNF-α, IL-1β, IL-6, and IL-12 (11, 61, 62). These mediators effectively activate Th1-type immune responses and promote the generation of reactive oxygen species (ROS) and nitric oxide (NO), thereby endowing M1-like macrophages with potent antimicrobial, antitumor, and debris-clearing capacities. M2-like macrophages are characterized by high expression of CD206 and CD163. They secrete anti-inflammatory and reparative cytokines such as IL-10 and TGF-β, along with chemokines including CCL17, CCL18, and CCL22 (63, 64). Their primary functions include suppressing inflammation, promoting tissue repair and remodeling, mediating angiogenesis, clearing apoptotic cells, and regulating immune tolerance.

Under physiological conditions, testicular macrophages predominantly exhibit an M2-like phenotype (65). This polarization state, through the secretion of regulatory cytokines such as IL-10 and TGF-β, suppresses excessive inflammatory responses and autoimmune attacks, thereby protecting post-meiotic germ cells that express neoantigens (66). Interstitial M2-like macrophages closely interact with Leydig cells and directly support testosterone synthesis via paracrine signals, such as 25-hydroxycholesterol (67). Peritubular M2-like macrophages contribute to the establishment of the spermatogonial stem cell niche by secreting factors such as CSF-1, thereby supporting the initiation of spermatogenesis (30). Under pathological conditions such as infection, autoimmunity, ischemia/reperfusion injury, or metabolic disorders, testicular immune homeostasis is disrupted (68). Various disease-associated factors drive resident macrophages to undergo aberrant polarization from an M2-like phenotype toward a pro-inflammatory M1-like state (42). In addition, a substantial number of peripheral blood monocytes with an M1-like bias are recruited into the testicular tissue, further increasing the proportion of M1-like macrophages. Disruption of the M1/M2 balance, particularly the excessive activation and accumulation of M1-like macrophages, represents a central mechanism underlying testicular inflammatory injury. M1-like macrophages release large amounts of TNF-α, IL-1β, and ROS, which can directly damage Leydig cells, disrupt the BTB, and induce germ cell apoptosis, ultimately resulting in defective spermatogenesis and male infertility (9, 13). Therefore, in inflammation-associated spermatogenic dysfunction characterized by excessive and sustained M1-like responses, therapeutic modulation of macrophage polarization may represent a potential strategy to restore macrophage homeostasis and facilitate inflammation resolution. However, this approach should not be oversimplified as merely increasing M2-like macrophage abundance, as excessive or prolonged M2-like activation may induce pathological tissue remodeling and fibrosis.

Accumulating evidence indicates that macrophages are highly plastic and that the traditional M1/M2 polarization framework should be presented with caution. This binary classification does not accurately capture the *in vivo* diversity of macrophages, particularly in specialized tissue microenvironments such as the testis. Macrophages respond in a coordinated manner to multiple signals, including developmental origin, anatomical localization, cytokines, hormones, metabolites, stromal-cell-derived cues, and disease stage (69–72). As a result, macrophages often display dynamic, continuous, and spatially dependent activation states rather than two discrete phenotypes. Multiple scRNA-seq studies in tumors have shown that markers traditionally associated with M1 and M2 macrophages are insufficient for distinguishing macrophage subtypes (73, 74). Similarly, testicular macrophages should not be regarded as a homogeneous M1 or M2 population. Instead, “M1-like” and “M2-like” should be considered operational descriptors of inflammatory, immunoregulatory, or reparative programs, not fixed lineage identities.

Macrophage ontogeny and activation state should be considered as two related but distinct dimensions. Under physiological conditions, resident testicular macrophages, including interstitial and peritubular macrophages, are long-lived tissue-adapted cells maintained within specialized testicular niches. These resident macrophages contribute to immune privilege, Leydig cell function, steroidogenesis, and the maintenance of the spermatogenic microenvironment. They are often enriched for immunoregulatory, tolerogenic, or M2-like features; however, this phenotype should be interpreted as a tissue-adapted functional state rather than a fixed M2 identity (12, 33). In response to infection, autoimmune inflammation, or tissue injury, resident macrophages may also acquire M1-like inflammatory or antigen-presenting programs (10, 42). Conversely, circulating monocytes can be recruited into the testis under pathological conditions, for example through chemokine pathways such as C-C motif chemokine ligand 2 (CCL2)/C-C motif chemokine receptor 2 (CCR2), and subsequently differentiate into monocyte-derived macrophages (75, 76). These recruited macrophages may display M1-like inflammatory features during the early phase of injury, but may later acquire M2-like reparative, remodeling, or even profibrotic programs during inflammation resolution and tissue repair (77–79).

Therefore, resident M2-like macrophages and monocyte-derived M2-like macrophages may share some M2-associated markers, such as CD206, IL-10, ARG1, or extracellular matrix (ECM) related genes, but they are not equivalent cell populations (80, 81). Resident M2-like macrophages mainly represent steady-state, tissue-adapted immunoregulatory cells involved in testicular homeostasis, whereas monocyte-derived M2-like macrophages usually arise in response to pathological insults and may participate in debris clearance, inflammation resolution, tissue remodeling, or fibrosis (31, 77). Similarly, resident M1-like and monocyte-derived M1-like macrophage states should not be regarded as equivalent: the former reflects context-induced activation of tissue-adapted macrophages, whereas the latter often reflects newly recruited inflammatory macrophages (80, 81).

Taken together, the testicular microenvironment may exert unique molecular and spatial regulatory mechanisms on macrophage activation states. Recognition of this complexity provides a phenotypic basis for future investigations into the roles of distinct testicular macrophage subsets in normal development and in the pathogenesis of spermatogenic dysfunction. Future studies should integrate lineage tracing, scRNA-seq, spatial transcriptomics, multiplex immunostaining, and functional validation to systematically elucidate the authentic functional states of testicular macrophages beyond the conventional M1–M2 dichotomy. Particular emphasis should be placed on their dynamic plasticity and state transitions within the local microenvironment, thereby uncovering their context-dependent roles and functional complexity in spermatogenesis.

## 4 Macrophage-mediated inflammatory remodeling of the testicular microenvironment in spermatogenic dysfunction

Under pathological stimulation, testicular macrophages exhibit polarization imbalance, expansion, and secretory reprogramming, thereby initiating and sustaining chronic local inflammation. This macrophage-driven response further remodels the inflammatory testicular microenvironment through complex intercellular crosstalk.

Inflammatory signals released by macrophages first act directly on two key somatic cell types in the testicular microenvironment: Sertoli cells and Leydig cells. Under inflammatory conditions, macrophages shift from homeostatic-supportive cells to detrimental regulators, thereby compromising their supportive effects on these cells (82, 83). As the function of key testicular somatic cells is impaired, the testicular tissue architecture they maintain undergoes marked remodeling, characterized by abnormal ECM turnover and interstitial fibrosis. During acute inflammation, recruited macrophages secrete matrix metalloproteinases (MMPs), such as MMP2 and MMP9, to degrade pre-existing ECM, thereby facilitating immune cell infiltration and tissue repair (84). However, excessive ECM degradation may disrupt the seminiferous tubule basement membrane and BTB homeostasis, leading to impaired Sertoli cell junctions and an imbalanced spermatogenic microenvironment. At sites of tissue injury, macrophages release profibrotic mediators, such as TGF-β and platelet-derived growth factor, which induce fibroblast proliferation and activation, thereby directly promoting fibrosis (75). Macrophage-driven inflammation may also promote mast cell accumulation around seminiferous tubules; mast cell-derived tryptase further enhances fibroblast proliferation and collagen synthesis, leading to peritubular fibrosis (85–87). Excessive fibrotic deposition ultimately results in seminiferous tubule distortion, basement membrane thickening, and increased tissue stiffness.

CCR2 is an important regulator of testicular fibrosis and ECM remodeling, primarily through macrophage-mediated mechanisms. Peng et al. (75) generated CCR2-deficient mice and employed an experimental autoimmune orchitis (EAO) model for mechanistic investigation. They found that in wild-type EAO mice, profibrotic macrophages expressing fibronectin and collagen I were markedly expanded, reaching approximately 117-fold and 70-fold of control levels, respectively. In contrast, in CCR2-deficient EAO mice, the expansion of these profibrotic macrophages was significantly suppressed, with their numbers reduced to approximately one-seventh of those observed in wild-type EAO mice. In addition, the ECM-related factors MMP2 and MMP14 were significantly upregulated in the testes of EAO mice, whereas their levels were markedly reduced in CCR2-deficient mice.

The combined effects of cellular dysfunction and structural damage ultimately lead to the breakdown of testicular immune privilege. Disruption of Sertoli cell tight junctions at the cellular level, together with fibrotic thickening of the basement membrane at the structural level, compromises the integrity of the BTB and impairs its physical barrier function. As a result, post-meiotic germ cells in the seminiferous tubules may be exposed to the immune system, triggering autoreactive T cells and an immune attack on germ cell antigens, which can start or worsen autoimmune orchitis (11). Meanwhile, reduced local anti-inflammatory factors, increased pro-inflammatory cytokines, and decreased immunosuppressive molecules, such as testosterone and activin A, further shift the microenvironment from an immunosuppressive to a pro-inflammatory state. This creates a vicious cycle in which persistent inflammation promotes germ cell apoptosis, while apoptotic cell debris further activates macrophages and recruits infiltrating monocytes, thereby amplifying the inflammatory response.

Together, these multi-level alterations drive inflammatory remodeling of the testicular microenvironment, making it unable to support normal spermatogenesis. Consequently, germ cell proliferation, meiosis, and differentiation are impaired, ultimately resulting in spermatogenic dysfunction.

## 5 Macrophage alterations in different types of spermatogenic dysfunction

### 5.1 Macrophage dysregulation constitutes a key pathogenic mechanism in NOA

Macrophage-driven inflammation and immune homeostatic imbalance constitute a key pathogenic mechanism in NOA. Goluza et al. (88) conducted histological and immunohistochemical analyses on testicular biopsy specimens from patients diagnosed with NOA (n = 108) and OA patients (n = 12). They found that macrophages were significantly increased in the testes of NOA patients and exhibited an aberrant distribution pattern. Further, Zheng et al. (15) integrated multiple public datasets of NOA for bioinformatic analyses and validated their findings using real-world clinical samples, including testicular biopsies from NOA patients (n = 16) and normal controls (n = 6). They found that both M1 and M2 macrophage infiltration were significantly increased in the testes of NOA patients compared with normal samples. This indicates that NOA testicular tissue is not in a purely pro-inflammatory state, but rather exhibits a highly activated and dysregulated immune microenvironment. This environment is marked by the concurrent activation of both pro-inflammatory pathways (e.g., IL-23, IL-6, and IL-1), and anti-inflammatory pathways (e.g., IL-10, IL-4, and TGF-β) (15, 16). Elevated levels of pro-inflammatory cytokines released by M1 macrophages directly inhibit spermatogonial stem cell proliferation and induce germ cell apoptosis (89, 90). Additionally, inflammatory mediators undermine the integrity of the BTB. Once this barrier is compromised, deleterious substances and inflammatory factors can more easily infiltrate the lumen of the seminiferous tubules, directly attack developing germ cells and establishing a vicious cycle (91, 92). Furthermore, the dysregulation of macrophages indirectly disrupts spermatogenesis by impairing the homeostasis and function of Sertoli and Leydig cells. The chronic inflammatory microenvironment, resulting from imbalanced macrophage polarization, can directly damage Sertoli cells (93, 94). This process is closely associated with significant disturbances in fatty acid metabolism (95). Research indicates that in the testes of patients with NOA, there is a reduction in the proportion of Sertoli cells and/or a compromise in their function (96). In NOA, the interaction between interstitial macrophages and Leydig cells transitions from a supportive to a deleterious state. Pathologically activated macrophages exert negative regulation on Leydig cells through paracrine mechanisms, resulting in morphological abnormalities (e.g., vacuolization), and functional impairment (88).

### 5.2 Macrophage accumulation in OA and its potential impact on spermatogenesis

In OA, macrophages primarily play a secondary and reactive role, in contrast to their function as primary pathogenic drivers in NOA. Chronic obstruction can lead to increased intraluminal pressure within the seminiferous tubules, fluid retention, and may induce local ischemia or mild inflammation, thereby recruiting and activating macrophages to perform clearance and repair functions (97). Thus, macrophage accumulation is not an initiating factor in OA, but rather a secondary pathophysiological response to prolonged obstruction. Studies on vasectomy have demonstrated that prolonged obstruction of the vas deferens induces epididymal inflammation, accompanied by increased macrophage infiltration and altered distribution (98, 99). In addition, Chen et al. (100) through scRNA-seq of testicular samples from OA patients and normal controls, found that contractility-related genes (e.g., MYH11) were upregulated in peritubular myoid cells (PTMCs), whereas negative regulators of contraction (e.g., GATA4) were downregulated. This suggests that PTMCs may remain in a sustained contractile state in response to increased intratubular pressure, attempting to counteract fluid retention and pressure-induced damage caused by obstruction. More importantly, the proportions of round and elongating spermatids were significantly reduced in OA testes, whereas undifferentiated spermatogonia were markedly increased, indicating a spermatogenic arrest prior to the early spermatid stage in OA patients. Although macrophage accumulation is not the initiating event in obstruction, their abnormal distribution and increased numbers during later stages of OA may exacerbate the deterioration of the spermatogenic microenvironment, thereby contributing to impaired spermatogenesis. Theoretically, it cannot be excluded that, under conditions of long-standing obstruction, some patients may gradually develop spermatogenic dysfunction and even exhibit NOA-like features. However, this speculation still needs to be validated in humans. If such cases are inadvertently included in NOA cohorts, the observed “macrophage abnormalities” may be misattributed to primary pathogenic mechanisms of NOA, whereas they may in fact represent a shared downstream consequence of chronic obstruction. This could lead to an overestimation of the initiating role of macrophages in NOA and obscure the fundamental differences between OA and NOA at early disease stages.

### 5.3 Macrophage dysregulation in oligozoospermia, asthenozoospermia, and teratozoospermia

Macrophage dysregulation can result in a diminished sperm count, decreased motility, and abnormal sperm morphology. Chronic, low-grade testicular inflammation is commonly observed in individuals with oligozoospermia (13). This condition may be closely linked to an increased abundance of macrophages and elevated levels of pro-inflammatory mediators. In a case of oligoasthenozoospermia with concurrent epididymitis, macrophages and dendritic cells were noted to directly capture and phagocytose spermatozoa within the epididymal tubular lumen (101). Furthermore, inflammation and oxidative stress mediated by macrophages can lead to mitochondrial dysfunction in sperm, thereby reducing ATP production and directly impairing the energy supply necessary for flagellar motility (102–104). Ubiquitin-specific peptidase 2 (USP2) is an important member of the deubiquitination system (105). Bedard et al. (106) demonstrated that sperm from male *Usp2* knockout mice exhibited significant motility impairments. Further, Hashimoto et al. (107) constructed myeloid cell–specific *Usp2* knockout mouse model and conducted investigations. They found that following cryopreservation and thawing, sperm from these mice displayed markedly reduced motility, capacitation, hyperactivation, and *in vitro* fertilization capacity. Mechanistically, the deletion of USP2 leads to diminished expression of granulocyte-macrophage colony-stimulating factor (GM-CSF). This reduction in GM-CSF is directly linked to mitochondrial dysfunction, motility impairments, and reduced fertilization capacity in sperm (50, 87). Macrophage activation is associated with increased ROS production. The sperm plasma membrane, which is rich in polyunsaturated fatty acids, is highly susceptible to ROS-induced damage. Elevated ROS levels can cause membrane lipid peroxidation, DNA damage, and protein dysfunction, resulting in abnormal sperm morphology (e.g., cytoplasmic retention and head deformities) (51). Moreover, abnormal morphology and immature sperm may further contribute to ROS accumulation, exacerbating oxidative injury (108).

In summary, irrespective of whether the initiating factors are infections, metabolic disorders, genetic defects, or aging, these insults ultimately lead to the aberrant activation and pro-inflammatory polarization of macrophages within the testis or reproductive tract. This disrupted immune microenvironment serves as a common pathological foundation for conditions such as oligozoospermia, asthenozoospermia, teratozoospermia, and more severe manifestations like azoospermia. Consequently, modulating macrophage polarization and mitigating pathological inflammation may provide potential therapeutic strategies for the treatment of spermatogenic dysfunction, and offer new avenues and targets for intervention.

## 6 Therapeutic strategies targeting macrophages for the treatment of spermatogenic dysfunction

### 6.1 Regulation of macrophage polarization

The modulation of macrophage polarization is regarded as a pivotal therapeutic strategy for addressing male infertility linked to spermatogenic dysfunction. The primary objective is to reprogram aberrantly activated, pro-inflammatory macrophages within the testis toward phenotypes with anti-inflammatory, immunosuppressive, and supportive functions. Such reprogramming promotes the restoration of the disrupted spermatogenic microenvironment. Guo et al. (109) found that the activation of the ring finger protein 8 (RNF8)/optineurin (OPTN)/lysine demethylase 6A (KDM6A) axis has the potential to inhibit M1-like macrophage polarization. RNF8 facilitates the degradation of KDM6A through the autophagy–lysosome pathway by modulating the activity of OPTN. Importantly, the expression of *KDM6A* is positively correlated with the pro-inflammatory cytokines IL6 and IL12a, whereas it is negatively correlated with the anti-inflammatory cytokine IL10 (110). In addition, insulin-like peptide 3 (INSL3) secreted by Leydig cells acts on macrophages via its receptor relaxin family peptide receptor 2. Subsequent, activation of the cAMP/PKA and Akt/mTOR signaling pathways promotes IL-4–induced M2 polarization (111). Therefore, INSL3 supplementation or pharmacological activation of INSL3-related signaling pathways may help maintain or restore the anti-inflammatory macrophage phenotype under inflammatory conditions. Similarly, in a mouse model of orchitis, exogenous administration of corticosterone activates the AMP-activated protein kinase (AMPK) signaling pathway, thereby enhancing fatty acid oxidation and subsequently promoting polarization of testicular macrophages toward an anti-inflammatory M2-like phenotype (112). In addition, targeting NOD-, LRR-, and pyrin domain-containing protein 3 (NLRP3) (e.g., thymoquinone) suppresses the NLRP3/caspase-1/IL-1β signaling axis, thereby promoting macrophage M2 polarization and alleviating tissue injury (113). S100 calcium-binding protein A9 (S100A9) has been shown to induce M2 polarization of macrophages through activation of the PI3K/Akt signaling pathway (114). Xu et al. (90) demonstrated that inflammation-derived testicular exosomes enriched in microRNA-155-5p (miR-155-5p) are internalized by macrophages and promote their polarization toward a pro-inflammatory M1-like phenotype. Furthermore, *in vivo* experiments showed that loading miR-155-5p inhibitors into these exosomes attenuated M1 polarization in the mouse testis, reduced pro-inflammatory cytokine secretion, and partially restored testicular immune homeostasis.

It should be emphasized that M2-like macrophages are not invariably beneficial in the context of a testicular inflammatory microenvironment. Although M2-like macrophages are generally associated with inflammation resolution, efferocytosis, immunoregulation, and tissue repair, their effects are highly context-dependent. During the early resolution phase of acute injury, an appropriate M2-like response may help terminate excessive inflammation and support restoration of the spermatogenic microenvironment. However, excessive or persistent M2-like activation may become detrimental, particularly under chronic inflammatory conditions, by promoting ECM deposition, pathological tissue remodeling, and fibrosis. This is especially relevant in the testis, where fibrotic changes in the interstitial or peritubular compartment may impair the local niche required for normal spermatogenesis. Therefore, macrophage-targeted therapies should aim to restore a balanced, stage-appropriate response rather than simply increasing M2-like macrophages, ensuring inflammation resolution without driving fibrotic remodeling.

### 6.2 Targeting macrophage-associated signaling pathways

Targeting macrophage-associated inflammatory signaling pathways enables precise intervention at specific molecular switches that drive macrophage inflammatory functions. Zhu et al. (115) demonstrated that peritubular macrophages in middle-aged men and mice experience pro-inflammatory and glycolytic metabolic reprogramming mediated by chemokine-like receptor 1 (CMKLR1) signaling, leading to impaired spermatogenesis. In mouse model, administration of the CMKLR1 antagonist peptide P12C5 counteracts this reprogramming, thereby restoring immunometabolic equilibrium and recovering spermatogenic function. Targeting pro-inflammatory signaling pathways, such as the nuclear receptor subfamily 2 group C member 2 (NR2C2)/NF-κB axis, calcium-sensing receptor (CaSR)/NLRP3 signaling, and Gasdermin D (GSDMD), has been shown to ameliorate testicular inflammation. NR2C2 activate NF-κB–dependent transcription, leading to increased production of inflammatory cytokines (e.g., IL-1β and IL-6). *In vitro* studies show that macrophage-specific NR2C2 knockdown partially alleviates the inflammation-mediated inhibition of the proliferation of spermatogonia (116). The CaSR/NLRP3 signaling pathway facilitates the maturation and secretion of IL-1β, which in turn directly inhibits testosterone synthesis (117). Intra-testicular administration of the CaSR antagonist NPS2143 in rats disrupts this pathway, reduces inflammation, and partially restores testosterone levels (118). GSDMD promotes pyroptosis, inflammatory responses, and antigen presentation, thereby exacerbating tissue damage and T-cell activation. In mouse model, treatment with the GSDMD inhibitor Dimethyl fumarate alleviates testicular inflammation and improves sperm quality (119). In addition, the activation of CCAAT enhancer binding protein beta (Cebpb) may upregulate the expression of critical genes involved in testosterone synthesis and enhance the local steroidogenic support offered by macrophages, thereby exerting an indirect influence on spermatogenesis (47). Given the cooperative roles of mast cells and macrophages in inflammatory responses, mast cell stabilizers such as ketotifen have also been considered promising therapeutic options. Post-varicocelectomy, ketotifen has demonstrated efficacy in enhancing sperm parameters and chromatin integrity, as well as in elevating pregnancy rates (120). However, the indirect effects of ketotifen on macrophages remain unclear. Further mechanistic studies are required to determine whether ketotifen regulates macrophage activation, polarization, or macrophage–mast cell interactions in spermatogenic dysfunction.

### 6.3 Stem cell therapies

The theoretical basis of stem cell transplantation for the treatment of male spermatogenic dysfunction lies in the capacity of exogenous stem cells to act as regenerative units or supportive components within damaged testicular tissue. Through these roles, they may reconstruct or repair disrupted spermatogenic processes. Current therapeutic strategies can be broadly divided into two primary approaches. The first approach, germ cell lineage–specific strategy, involves the transplantation of healthy spermatogonial stem cells into the seminiferous tubules to reinitiate spermatogenesis *in situ* (121). The second approach primarily involves the transplantation of stem cells related to the testicular microenvironment, including mesenchymal stem cells (MSCs) and stem Leydig cells (SLCs) (122). This strategy aims to restore crucial hormonal support through cellular replacement and to influence the activation state of local macrophages, thereby restructuring the immune microenvironment (123).

MSCs, mainly including bone marrow–derived MSCs and adipose tissue–derived MSCs. They have been extensively investigated for the treatment of various spermatogenic dysfunction owing to their robust multilineage differentiation potential, paracrine activity, and immunomodulatory properties. MSCs secrete anti-inflammatory cytokines (e.g., IL-10 and TGF-β), which directly facilitate macrophage M2 polarization (51, 124, 125). By modulating macrophage activity, MSCs can also mitigate the excessive production of profibrotic factors (e.g., TGF-β1), thereby preventing interstitial fibrosis of the testis and disrupting the detrimental cycle between inflammation and fibrosis (126). In various animal models of testicular injury, the transplantation of MSCs has demonstrated efficacy in reducing testicular inflammation and restoring spermatogenic function (127–130). Furthermore, a clinical trial involving 87 patients with NOA reported that intratesticular injection of MSCs led to the detection of spermatozoa in the ejaculate of 18 patients (20.7%) at different follow-up intervals (131). However, the overall efficacy remains modest, and the long-term safety profile has yet to be clearly established.

SLCs represent a stem cell–based therapeutic approach with a more clearly defined macrophage-targeting mechanism. Transplanted SLCs can directly regulate macrophage function through a unique mechanism known as “mitochondrial transfer”, thereby suppressing testicular inflammation, promoting tissue repair, and restoring fertility (132, 133). In rodent models of testicular injury or aging, activated macrophages produce excessive levels of ROS. SLCs are capable of sensing these injury-associated signals and, via the transient receptor potential melastatin 7 (TRPM7) ion channel, transferring their functional mitochondria directly to surrounding macrophages (134). Macrophages that acquire healthy mitochondria exhibit improved metabolic activity and functional status, leading to a marked attenuation of inflammatory responses. Although SLCs therapy has made some progress in improving spermatogenesis in animal models, the mechanism of SLC-mediated “mitochondrial transfer” still requires further validation in testicular tissues (132, 134–136). In particular, the precise role of TRPM7-mediated mitochondrial transfer in testicular immune microenvironment remains unclear. Moreover, whether this mechanism exists in human testicular tissue remains to be further confirmed. **Table 1** summarize the proposed therapeutic targets and strategies, including their mechanisms, signaling pathways, levels of evidence, key findings, and safety assessments.

**Table 1:** Macrophage-related therapeutic targets and intervention strategies for spermatogenic dysfunction.

Author	Year	Intervention strategy or target	Mechanism/signaling	Evidence level	Key findings	Safety assessment
Zhou et al. (111)	2025	INSL3	cAMP/Akt/mTOR	In vitro	INSL3 promotes M2-like macrophage polarization	Not evaluated
Guo et al. (109)	2025	RNF8	RNF8/OPTN/KDM6A	Mouse model	The RNF8/OPTN/KDM6A axis suppresses M1-like macrophage polarization	Not evaluated
Zhang et al. (112)	2020	Corticosterone	Corticosterone-mediated AMPK activation	Mouse model	Corticosterone activates AMPK to promote M2 polarization	Not evaluated
Fan et al. (114)	2021	S100A9	S100A9/PI3K/Akt	Mouse model	S100A9 promotes M2-like macrophage polarization	Not evaluated
Elhessy et al. (113)	2025	NLRP3	NLRP3/Caspase 1/IL-1β	Rat model	The NLRP3/caspase-1/IL-1β axis promotes M2-like macrophage polarization	Not evaluated
Zhu et al. (115)	2026	CMKLR1	CMKLR1-mediated pro-inflammatory glycolysis	Mouse model	Blockade of CMKLR1 or high-intensity interval training restores anti-inflammatory metabolism and rescues spermatogenesis	Not evaluated
Ma et al. (119)	2024	GSDMD	GSDMD-mediated inflammation and antigen presentation	Mouse model	Pharmacological inhibition of GSDMD alleviated testicular inflammation	Not evaluated
Min et al. (116)	2023	NR2C2	NR2C2/NF-κB	Mouse model	NR2C2 inhibition in macrophages attenuates inflammatory responses	Not evaluated
Su et al. (118)	2020	CaSR	CaSR/NLRP3 inflammasome	Mouse model	Macrophage CaSR activates the NLRP3 inflammasome pathway and promotes IL-1β secretion	Not evaluated
Xu et al. (90)	2023	Testis-derived exosomes	MiR-155-5p delivery	Mouse model	Exosomes loaded with miR-155-5p inhibitors attenuate M1-like macrophage polarization	Not evaluated
Duan et al. (47)	2024	Cebpb	Regulation of steroidogenic enzyme expression (Cyp11a1, Cyp17a1)	Mouse model	Cebpb regulates autonomous testosterone biosynthesis in testicular macrophages	Not evaluated
Alhefnawy et al. (131)	2024	MSCs	Anti-inflammatory cytokine secretion facilitating M2-like macrophage polarization	Clinical trial in NOA patients (n = 87)	Intratesticular MSCs injection improves spermatogenic outcomes in some NOA patients (20.7%)	No major short-term adverse effects; long-term carcinogenic risk remains unclear
Chi et al. (134)	2024	SLCs	Mitochondrial transfer to resident macrophages	Mouse model	SLCs suppress inflammatory responses via mitochondrial transfer to macrophages	Not evaluated

INSL3, insulin-like peptide 3; RNF8, ring finger protein 8; OPTN, optineurin; KDM6A, lysine demethylase 6A; AMPK, AMP-activated protein kinase; S100A9, S100 calcium-binding protein A9; NLRP3, NOD-, LRR-, and pyrin domain-containing protein 3; CMKLR1, chemokine-like receptor 1; GSDMD, Gasdermin D; NR2C2, nuclear receptor subfamily 2 group C member 2; NF-κB, nuclear factor kappa B; CaSR, calcium-sensing receptor; MiR-155-5p: microRNA-155-5p; Cebpb, CCAAT enhancer binding protein beta; MSCs, mesenchymal stem cells; NOA, non-obstructive azoospermia; SLCs, stem Leydig cells.

## 7 Current challenges in macrophage-related therapies

Modulating macrophage polarization to improve the spermatogenic microenvironment has emerged as an important exploratory strategy for treating inflammation-associated male infertility. Although this approach is conceptually more appealing, this approach also presents significant challenges. First, nearly all studies investigating targets related to macrophage polarization, including the RNF8, INSL3, AMPK, miR-155-5p, NLRP3, and S100A9, have been conducted exclusively in mouse models, with no human data currently available to support their clinical efficacy or safety. Given the fundamental differences between humans and animals in testicular macrophage biology, signaling pathway sensitivity, and pharmacokinetics, the translational potential of these targets in humans remains highly uncertain.

Second, testicular macrophages display considerable heterogeneity. ScRNA-seq have revealed the presence of multiple macrophage subpopulations within the testis that differ in both function and spatial localization (40, 137). These include pathological pro-inflammatory subsets that necessitate suppression, alongside regulatory subsets that are crucial for preserving immune privilege. Current intervention strategies are predominantly systemic and broad-spectrum, such as hormonal or small-molecule drug administration. This nonspecific modulation may inadvertently disrupt normal immune tolerance, potentially compromising physiological functions.

Moreover, limitations in delivery systems represent a major bottleneck. At present, there is a lack of tissue-targeting technologies capable of selectively delivering regulatory molecules (e.g. miRNA inhibitors) to testicular macrophages. Long-term modulation of macrophage polarization may also disrupt local immune homeostasis and entail unpredictable long-term risks. For example, epigenetic targets such as KDM6A exert effects across multiple cell types, and inappropriate regulation may induce off-target gene expression changes, thereby increasing the risk of tumorigenesis. Signaling pathways such as PI3K/Akt and AMPK are not only involved in inflammatory regulation but also play critical roles in cellular metabolism, apoptosis, and tissue homeostasis. Prolonged intervention in these pathways may affect other testicular cell types and potentially extend to systemic organs. Thus, achieving an optimal balance between facilitating tissue repair and maintaining immune and physiological homeostasis represents a critical challenge for the successful clinical translation of this strategy.

Compared with direct modulation of macrophage polarization, strategies targeting key macrophage-associated signaling pathways theoretically offer greater potential for precision intervention. However, the etiology of spermatogenic dysfunction is heterogeneous, making it difficult to achieve broadly applicable therapeutic efficacy by targeting a single pathway or molecule. Reported targets, such as CMKLR1, NR2C2 and GSDMD, are frequently linked to specific pathological contexts. The inflammatory mechanisms within the testis and their upstream activation pathways exhibit substantial variability across different etiologies, and there is currently a lack of clinically applicable biomarkers for precise patient stratification. Moreover, numerous proposed targets are integral regulators of systemic immune function. For example, while CCR2 is involved in promoting fibrotic remodeling in testicular tissue, it is also crucial for the recruitment of immune cells and the regulation of inflammatory responses. Thus, non-selective inhibition of CCR2 could undermine normal immune surveillance and elevate the risk of infection. Similarly, NF-κB and NLRP3 are central regulators of systemic immune homeostasis, cell survival, and metabolic adaptation (138, 139). Broad suppression of these pathways may impair host defense, disrupt anti-apoptotic signaling and mitochondrial function, and compromise tissue repair, thereby negatively impacting the energetically demanding process of spermatogenesis. Targeting macrophage Cebpb boosts testosterone production, but its effectiveness in inflammatory conditions is uncertain. Additionally, overactivation may disturb hormonal balance, and precise intervention methods are needed.

Stem cell therapies offer a promising strategy for reconstructing the testicular immune environment and promoting spermatogenic repair, but challenges related to safety, controllability, and clinical translation hinder their application. First, stem cell transplantation is associated with potential safety risks, such as tumor formation, ectopic differentiation, and immune rejection, which may be heightened in the immune-privileged testicular environment. Current evidence for the regulation of macrophage function through stem cell-mediated mitochondrial transfer has been derived exclusively from animal models. The existence of this mechanism in humans, as well as the ability of transplanted cells to effectively home to target tissues, survive, and exert functional effects, has yet to be substantiated by experimental data. Although MSCs have shown some effectiveness in clinical trials for certain patients with NOA, the overall success rate is still low, and the mechanisms involved are not well understood, hindering their widespread clinical use as a universal treatment option. Moreover, variability in stem cell sources and inconsistencies in preparation standards pose challenges to achieving stable and reproducible therapeutic outcomes. Although stem cell therapies can ameliorate the inflammatory microenvironment by modulating macrophage function, they simultaneously affect multiple cellular systems, including vascular cells, Sertoli cells, and Leydig cells. These complex network-level effects are difficult to predict and monitor, complicating the balance between therapeutic efficacy and adverse effects and substantially limiting the clinical safety and controllability of such approaches.

## 8 Future perspectives in macrophage-related therapies

Future research should concentrate on three pivotal dimensions: mechanistic refinement, intervention specificity, and the optimization of delivery systems. First, the establishment of mechanism-validation frameworks using human samples is of paramount importance. The majority of currently proposed regulatory mechanisms are derived from mouse models; however, the human testis presents significant differences in immune architecture, macrophage heterogeneity, and metabolic characteristics. Consequently, future studies should enhance multi-omics investigations of human testicular tissue, including single-cell omics, spatial transcriptomics, and proteomics, to elucidate the functional characteristics of distinct macrophage subpopulations under both physiological and pathological conditions.

Second, it is imperative to actively promote the advancement of precision-targeted intervention technologies. To overcome the limitations in specificity associated with existing regulatory strategies, it is essential to explore testicular macrophage-directed delivery systems utilizing nanomaterials, liposomes, or targeting peptides. These systems would facilitate subset-specific delivery and precise release of pharmacological agents or RNA-based therapeutics. Concurrently, the development of artificial intelligence-assisted drug screening and structure prediction methodologies is crucial for identifying regulatory factors that exhibit both high targeting specificity and favorable safety profiles. This approach will establish a robust foundation for the refinement of immunomodulatory interventions.

Moreover, future research should prioritize the establishment of stem cell production systems that are free of animal-derived components, exhibit low heterogeneity, and incorporate robust quality-tracking mechanisms. This focus aims to enhance therapeutic consistency and clinical applicability. Concurrently, genetic engineering techniques may be utilized to modify stem cells, thereby augmenting targeted delivery and directed immunomodulatory functions. Additionally, there is a need for comprehensive long-term follow-up studies employing human stem cells within three-dimensional testicular organoid systems or humanized mouse models. Such studies are essential to systematically assess the safety, stability, and durability of therapeutic effects, ultimately facilitating the transition from foundational research to fully controllable clinical applications.

## 9 Conclusion

**Figure 5:**
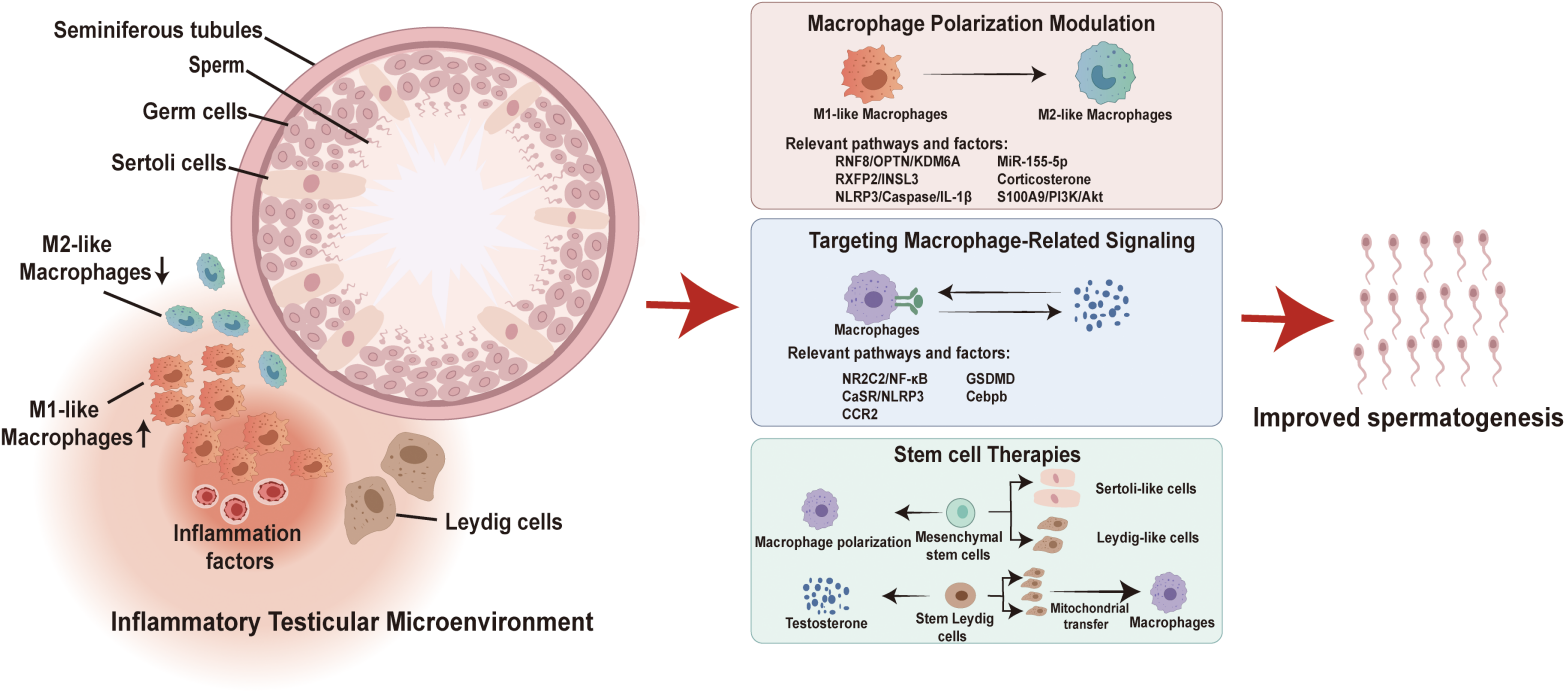
Macrophage-based therapeutic strategies for spermatogenic dysfunction. Currently, macrophage-targeted strategies for spermatogenic dysfunction can be broadly categorized into three approaches. Modulation of macrophage polarization promotes a shift from M1-like to M2-like phenotype, thereby restoring immune homeostasis. Targeting macrophage-related signaling pathways is mainly aimed at suppressing inflammatory responses and fibrotic progression. Stem cell therapies facilitate the restoration of spermatogenesis by rebuilding the testicular microenvironment. RNF8, ring finger protein 8; OPTN, optineurin; KDM6A, lysine demethylase 6A; RXFP2, relaxin family peptide receptor 2; INSL3, insulin-like peptide 3; NLRP3, NOD-, LRR-, and pyrin domain-containing protein 3; MiR-155-5p, microRNA-155-5p; S100A9, S100 calcium-binding protein A9; NR2C2, nuclear receptor subfamily 2 group C member 2; NF-κB, nuclear factor kappa B; CaSR, calcium-sensing receptor; CCR2, C-C motif chemokine receptor 2; GSDMD, Gasdermin D; Cebpb, CCAAT enhancer binding protein beta.

Testicular macrophages represent a central hub linking disrupted immune homeostasis to spermatogenic dysfunction. Aberrations in the number, spatial distribution, and functional phenotypes of testicular macrophages can induce chronic low-grade inflammation, disrupt immune privilege, and perturb hormonal homeostasis, thereby impairing the spermatogenic supportive microenvironment and ultimately leading to compromised spermatogenic function. In recent years, multiple potential therapeutic strategies targeting testicular macrophages have been proposed, including modulation of macrophage M1/M2 polarization, targeting macrophage-associated signaling pathways, and stem cell–based therapies (**Figure 5**). These novel approaches have exhibited differing levels of effectiveness in reducing inflammation and enhancing spermatogenesis in animal models. For example, supplementation with INSL3 signaling or corticosterone has been shown to drive testicular macrophages toward an M2-like phenotype in mice. Targeted inhibition of CMKLR1 signaling can suppress inflammatory responses and restore immunometabolic balance. In addition, transplanted SLCs can directly modulate macrophage function through a unique mechanism of mitochondrial transfer, thereby attenuating testicular inflammation and promoting tissue repair. However, most current studies remain confined to basic research and animal models, with a lack of high-quality human sample data and robust clinical evidence. The safety, efficacy, and translational feasibility of these approaches therefore require further validation. Future research should focus on elucidating the functional heterogeneity and dynamic regulation of testicular macrophages and accelerating the clinical translation of precision intervention strategies targeting these cells, thereby providing new therapeutic avenues for the treatment of male spermatogenic dysfunction.

## Glossary

NOA: non-obstructive azoospermia
OA: obstructive azoospermia
NF-κB: nuclear factor kappa B
ScRNA-seq: single-cell RNA sequencing
HSCs: hematopoietic stem cells
M-CSF: macrophage colony-stimulating factor
CSF-1: colony-stimulating factor-1
CSF-1R: colony-stimulating factor-1 receptor
RDH10: retinol dehydrogenase 10
BTB: blood–testis barrier
ROS: reactive oxygen species
NO: nitric oxide
CCL2: C-C motif chemokine ligand 2
CCR2: C-C motif chemokine receptor 2
ECM: extracellular matrix
MMP: matrix metalloproteinases
EAO: experimental autoimmune orchitis
PTMCs: peritubular myoid cells
USP2: ubiquitin-specific peptidase 2
GM-CSF: granulocyte-macrophage colony-stimulating factor
RNF8: ring finger protein 8
OPTN: optineurin
KDM6A: lysine demethylase 6A
INSL3: insulin-like peptide 3
AMPK: AMP-activated protein kinase
NLRP3: NOD-, LRR-, and pyrin domain-containing protein 3
S100A9: S100 calcium-binding protein A9
MiR-155-5p: microRNA-155-5p
CMKLR1: chemokine-like receptor 1
NR2C2: nuclear receptor subfamily 2 group C member 2
CaSR: calcium-sensing receptor
GSDMD: Gasdermin D
Cebpb: CCAAT enhancer binding protein beta
MSCs: mesenchymal stem cells
SLCs: stem Leydig cells
TRPM7: transient receptor potential melastatin 7
